# The Increased Vulnerability of Refugee Population to Mental Health Disorders

**Published:** 2018-02-28

**Authors:** Sameena Hameed, Asad Sadiq, Amad U. Din

**Affiliations:** 1 Department of Psychiatry and Behavior Science, University of Kansas School of Medicine, Kansas City, KS; 2 The Psychiatry and Therapy Centre, Dubai, United Arab Emirates

**Keywords:** Mental disorders, depression, PTSD, refugees, human migration

## Introduction

Around the world, the number of refugees displaced by war or violence reaches over 19 million. Rates of mental health disorders, such as anxiety disorders, post-traumatic stress disorder (PTSD) and depression were higher among refugee populations in comparison to the general population. This increased vulnerability has been linked to experiences prior to migration, such as war exposure and trauma. Additionally, anxiety and other mental health disorders can manifest due to stressors post-migration, such as separation anxiety and the added load of resettlement in a new country. In general, increased rates of these disorders remain prevalent in refugee populations long after resettlement; however, some studies have shown otherwise.^1^

In the Karenni refugees along the Burmese-Thai border, depression and anxiety rates (41% and 42%, respectively) were higher than the average rates of depression and anxiety among the general US population (7% and 10%, respectively).^2^ These rates have been linked to traumatic events like violence, harassment, and a lack of basic needs. Moreover, the mental health of refugees is thought to be distinct from the experiences of other traumatized populations, such as veterans and sexual assault victims, due to their unique traumatic experiences as well as acculturative stress that follows the resettlement process, which features entirely new settings, practices, and a lack of familiar support systems.^3^ Furthermore, this population showed a correlation of depression and anxiety disorders with post-resettlement hardships in regards to finding employment and adapting to a new environment culturally and linguistically. In another population, 82.6% of Cambodian refugees residing in a refugee camp on the Thailand-Cambodia border self-reported depression. Fifty-five percent were confirmed by the Hopkins Symptoms Checklist to have experienced symptoms of major depression.^2^

Symptoms of depression include changes in weight, sleep pattern, exhibiting a depressed mood for much of a day, a loss of interest in activities, lack of energy, feelings of worthlessness and guilt almost daily, lack of focus, and recurrent thoughts of death and suicide, which can include attempting or creating plans for suicide. Symptoms of PTSD include intrusion, avoidance, and hyperarousal. PTSD typically is associated with traumatic experiences. These traumatic events can include experiencing war, being held prisoner/hostage, torture and physical violence, death of a loved one, serious accidents/explosions, sexual harassment, and serious illness. Symptoms of generalized anxiety disorder include restlessness, irritability, fatigue, excessive worrying, having trouble relaxing, sleeping, and focusing.^4^

The current refugee demographic is a highly heterogeneous group, however, there has been an increase of refugees from Arabic speaking countries in recent years.^5^ Europe, in particular, has seen a large increase of asylum applicants from Arabic speaking countries, the most frequent being Syria (35.9% of applications) and Iraq (6.9% of applications). Despite the growth of Arabic speaking refugees, few studies have investigated the mental health of these populations in recent years. The large variations in results show that the refugee population is a diverse group. Complications in studies that inhibit direct comparison between refugee populations include the use of different psychometric instruments to measure mental health.^5^

Another factor that could promote the symptoms of PTSD, depression, and anxiety is acculturative stress. While trauma related to war negatively impacts mental health, the effects of acculturative stress on mental health among refugees resettled in Australia and Austria demonstrated that stress which accompanies the migration process can have similar effects.^6^ One cause of these stressors is acculturation, the process of integrating into a new culture while also maintaining one’s origin culture and identity. This process is dependent on the attitudes of both the migrant and host groups. There are inconsistencies present in existing studies investigating the effects of acculturation on mental health; however, acculturative stress in migration has been identified as a mental health risk factor.^6^

The purpose of this review was to investigate the relationship between refugee populations and their increased vulnerability to post-traumatic stress disorder, depression, and anxiety disorders. This study also examined the factors before, during, and after the migration process associated with increased vulnerability of refugees to mental health disorders.

## Refugee Health

In a particular population of Yazidi refugees, Nasiroglu et al.^7^ determined the frequency of post-traumatic stress disorder and depression among children and adolescents and examined the possible differences in experience and diagnosis between males and females. Big differences existed in the resulting diagnosis between children and adolescents. Children generally had fewer problems with mental illnesses than adolescents, who may have increased stress related to having more siblings. Adolescents had more siblings, on average, than children. Other risk factors for depression, in particular, included having older parents, being female, and witnessing someone undergoing a violent or fatal situation. In terms of gender, females of both the children and adolescent groups were significantly more likely to have an established diagnosis, as compared to males, who in general, did not have one.

To examine the mental health of Yazidi children and adolescents further, Ceri et al.^8^ investigated the presence of psychiatric disorders immediately following forced migration. Various disorders, not only PTSD, had manifested in the refugee population within the early days of resettlement. Children who experienced forced migration exhibited more behavioral and emotional problems than children who had not experienced such trauma. Following forced migration, children were observed to be very shy after their arrival to the camp and avoided contact with other children. Additionally, they communicated fears of being captured and generally did not feel safe in their new environment. Most children also had difficulty sleeping. Over one-third of the children were diagnosed with depressive disorder.

Factors that could be associated with psychiatric symptoms and disorders were torture and other traumatic events.^9^ Civilians in war-zones typically experienced at least one traumatic event due to war, and war refugees often were subjected to torture. Among Syrian Kurdish refugees, there were positive correlations between PTSD symptoms and traumatic events such as being forced to flee one’s country, witnessing violence, and confinement due to violence. Moreover, while males were more likely to experience trauma, females were more likely to have symptoms of PTSD. However, Syrian Kurdish refugees in the Kurdistan region of Iraq displayed no significant difference in the prevalence of PTSD among males and females, which may be a result of cultural differences.^9^

Refugee populations who have experienced traumatic events often are vulnerable to increased symptoms if they experience another stressful event.^10^ Thus, it has been investigated whether new traumatic or stressful events affect mental health of an already PTSD diagnosed individual. Schock et al.^10^ studied refugees from Iran, the Balkan region, and Turkey. All participants were diagnosed with PTSD. Groups that experienced a new significant life event displayed increased avoidance behavior. Such behavior may be a mechanism for these individuals to avoid re-experiencing their past trauma. Additionally, stressful life events affected symptoms more than traumatic life events. Overall, new significant life events resulted in a significant increase in PTSD symptoms, especially avoidance.

Furthermore, refugees diagnosed with PTSD often were diagnosed with secondary psychotic features as well. Nygaard et al.^3^ found 74 of 181 refugees (41%) diagnosed with PTSD were identified to have secondary psychotic features. These secondary psychotic features included hallucinations and delusions, and the impact of these features can make PTSD with Secondary Psychotic features (PTSD-SP) a burdening disorder. Refugees are uniquely vulnerable to developing secondary psychotic features with PTSD, as these features are assumed to manifest because refugees usually are subjected to more long-term trauma than other PTSD patients. Moreover, refugee populations often lack familiar support systems as they seek asylum abroad to escape their threatening situations, exacerbating the problem.

## Prior to Migration

Prior to the migration process, there are environmental factors that can be associated with the development of mental disorders. In Ethiopian immigrants and refugees, rates of depression were significantly higher among individuals who experienced pre-migration trauma as well as internment in a refugee camp.^11^ Other factors, like witnessing death in a family and lacking resources such as water, shelter, and food, were associated with depression. Individuals who experienced more traumatic events were more vulnerable to depression, as trauma can lead to hopelessness and a loss of interest in activities. In North Korea, war and organized violence are not the primary reason for individuals to seek asylum, rather they often are trying to escape political oppression.^12^ Nevertheless, the traumatic experiences, such as torture, violence, imprisonment, and witnessing death, are shared.

In a group of North Korean refugees, insomnia, often associated with depressive and post-traumatic stress symptoms, was higher in those individuals who had experienced traumatic events prior to migration.^12^ These findings suggested that development of refugee insomnia could be associated with these traumatic experiences. A study of Syrian refugees in Turkey found that other factors could contribute to the development of PTSD, like being diagnosed with a psychiatric disorder in the past or having a family history of psychiatric disorder, along with experiencing trauma.^13^ Refugees face major obstacles to meet health care needs, along with trauma and prior diagnoses, while in war zones or areas affected by natural disasters.

## During Migration

During migration, there are other stressors that can be associated with depression and anxiety. Stress can be from an uncertainty in the future, as is typical of asylum seekers.^14^ In two Danish asylum centers, the mental health of rejected Iraqi asylum seekers was evaluated. In this group, the prevalence of anxiety symptoms was 94% and depression symptoms had a prevalence of 100%. The lengths of stay in the asylum centers, as well as the number of traumatic events, were thought to be risk factors associated with psychological distress.

Among those in refugee camps, daily stressors can exacerbate mental problems, such as lacking basic necessities, restricted movement, and continued concern for safety, as refugee camps are only short-term solutions.^15^ Consistency in the life of refugees can ease mental distress. For example, the prevalence of PTSD was lower than expected in a group of Syrian child refugees, perhaps because these children travelled with at least one parent, transferring a crucial part of the child’s psychosocial environment. Therefore, having a parent accompany children during travel in the migration process could be a protective factor that can reduce post-traumatic stress rates among some children. Additionally, a successful flight during migration was associated with creating feelings of hope for the future.^16^ However, the anxiety of the parent accompanying the child can also influence the child’s own anxiety, therefore, the presence of a parent may not always be favorable especially if parents have mental distress.^17^

## Post-Migration

Often, depression among refugees has long-term effects. A study of Guatemalan refugees in Mexico found a 38.8% lifetime prevalence of depression.^11^ Karenina refugees settled on the Thai-Burma border had a lifetime depression prevalence of 41.8%. Post-migration stress can be related to feelings of insecurity. A group of North Korean refugees settled in South Korea felt unsafe due to a fear of being arrested and deported back to North Korea.^12^ Post-migration mental distress also has been associated with acculturative stress. Refugees were about ten times more likely to have PTSD than the host country’s general population, illustrating that the mental distress in refugee populations does not disappear after resettlement.^18^ These PTSD rates were among 7,000 refugees resettled in western countries. The comparison of refugees with the general population may not be reflective of the whole picture, with need to compare refugee rates with other populations including veterans and/or domestic violence victims in future studies.^18^

Acculturation is the process of integrating oneself into a new culture while maintaining one’s origin culture and identity.^6^ This process can create a considerable amount of stress for new refugees trying to restart lives in new countries, often resulting in anxiety and depression, as well as the exacerbation of post-traumatic stress. Acculturative stress is based on the demands of immigration experience. It is related to experiences that cause stress among immigrants and refugees. These include unfamiliarity with daily tasks, difficulties in finding employment, learning the host country’s language, discrimination, and a feeling of not belonging in one’s new environment. As an example of overcoming language barriers and its effect on mental health, Bosnian refugees living in Australia reported significantly more stress in terms of accommodating to the host language than Bosnian refugees living in Austria. Acculturative stress affects mental health based on the social atmosphere a refugee experiences in a host country, indicated by immigration policies and the general attitude of the host society towards refugees and different cultures.

## Discussion and Conclusion

Refugee populations have an increased vulnerability to post-traumatic stress disorder, depression, and anxiety due to their exposure to traumatic experiences prior to migration.^12^,^13^ Prior to the migration process, refugees often experience trauma from organized violence and political oppression, which can include the death of a loved one, torture, imprisonment, witnessing public executions, and lacking basic necessities.^12^,^13^ Other risk factors prior to migration include previous diagnoses of psychiatric disorders in oneself and/or family members.^14^ The development of such disorders can happen regardless of age; however, some age groups may experience more intense symptoms than others. Children in particular can develop behavioral and emotional problems as a result of certain traumatic experiences they may face, including forced migration.^8^ However, adolescents were more likely to have PTSD, which could be related to risk factors such as having more siblings or older parents, among others.^7^

Studies varied in regards to showing differences in the manifestation of mental distress between males and females. Nasiroglu et al.^7^, however, showed being female as a risk factor for depression. Females were more likely than males to have an established mental health diagnosis. In relation to PTSD, women were more likely to exhibit PTSD symptoms; however, this has not been consistent when the prevalence of PTSD was investigated among some Syrian Kurdish refugees, possibly due to culture differences.^9^ Nevertheless, being female generally was associated with increased prevalence of mental distress.

During the migration period, there were several factors that contributed to mental distress, such as lingering feelings of unsafety and uncertainty in the future. Prevalence rates of depression and anxiety among refugee populations who were denied asylum were high.^14^,^15^ A protective factor that helps when migrating with children is maintaining some aspects of a refugee’s previous environment, such as ensuring the child travels with at least one parent.^16^

Post-migration can include many difficulties that can cause mental distress to be worsened and/or have a long-term presence of mental health symptoms. A common factor associated with mental distress post-migration is acculturative stress, often experienced by refugees and immigrants.^6^ Experiences that result in acculturative stress include unfamiliarity with daily tasks, overcoming language barriers, and facing discrimination, among others. Acculturative stress often is unique to one’s environment because of the attitudes of the host country and whether certain changes in environment, such as language, are great. Not only are refugee populations vulnerable to PTSD, but they also face secondary features with their PTSD, increasing the burden of the mental disorder. These features can include hallucinations and delusions. Refugees are uniquely vulnerable to these secondary features because of their more long-term trauma. They are thrust into unfamiliar environments and lack familiar support systems. Consequently, refugees with PTSD are likely to experience secondary psychotic symptoms as well.^10^

This review article highlighted the higher prevalence rates of mental health disorders among refugees, especially depression, anxiety, and PTSD, who have experienced trauma and forced migration from their regions/countries. It underscored the importance of managing mental health scars and disorders with great empathy and higher level of care. Possible scenarios to help include, but are not limited to, involving family members in their care, language interpreters, being patient with them, and establishing an inclusive environment that accounts for the psycho-socio-cultural aspects of refugee lives.^6^,^18^ Comparing refugees with the general population may not be reflective of whole picture. Therefore, further need exists to compare refugee mental health rates with other populations including veterans, domestic violence victims, and/or other violence victims in future studies.

There are few studies available on mental health issues in the refugee population, possibly due to a lack of funding in this clinical arena. Moreover, few studies have mentioned potential errors in reporting data due to inability of the refugees to report their symptoms accurately under moderate to severe mental distress. More studies are needed to examine the increased vulnerability of refugee populations to mental health disorder and management guidelines to integrate them better and more fully into a new host society.
